# Recombinant *α*-Klotho Protein Alleviated Acute Cardiorenal Injury in a Mouse Model of Lipopolysaccharide-Induced Septic Cardiorenal Syndrome Type 5

**DOI:** 10.1155/2019/5853426

**Published:** 2019-06-11

**Authors:** Xi Liu, Yangyang Niu, Xiaoqin Zhang, Yingying Zhang, Ying Yu, Jieli Huang, Jiangtao Li, Chen Yu

**Affiliations:** The Department of Nephrology, Tongji Hospital, Tongji University School of Medicine, Shanghai, China

## Abstract

**Background and Aims:**

Klotho is an aging-suppressor gene mainly expressed in the renal tubules. The klotho gene encodes the *α*-klotho protein, which has many functions. Previous studies have found that *α*-klotho protein has a cardiorenal protective function. *α*-Klotho deficiency renders the kidney more susceptible to injury and results in cardiovascular calcification and left ventricular hypertrophy in chronic kidney disease. However, the role of *α*-klotho in acute heart injury and acute kidney injury with sepsis remains unknown. This study aimed to investigate the effects and mechanisms of *α*-klotho in septic cardiorenal injury.

**Methods:**

Male 8-week-old C57BL/6 mice were randomly assigned to the control group, lipopolysaccharide (LPS; 10 mg/kg) group, LPS (10 mg/kg)+*α*-klotho (0.01 mg/kg) group, and LPS (10 mg/kg)+*α*-klotho (0.02 mg/kg) group. Recombinant *α*-klotho was intraperitoneally injected an hour before LPS injection. Mice were euthanized at 24 h after LPS injection. The serum troponin, brain natriuretic peptide (BNP), neutrophil gelatinase-associated lipocalin (NGAL), and creatinine levels were measured in all groups at 24 h. Biomarkers of mice heart apoptosis, inflammation, oxidative stress, and endoplasmic reticulum stress, such as caspase-3, interleukin 1 (IL-1), reactive oxygen species (ROS), and glucose-regulated protein 78 (GRP78), were also measured.

**Results:**

*α*-Klotho was mainly expressed in mice kidneys and was undetectable in the control mice hearts. *α*-Klotho substantially decreased after LPS injection. In the LPS group, the serum troponin levels significantly increased as early as 6 h (*p* < 0.05) after LPS injection, while the BNP, NGAL, and creatinine levels significantly increased at 24 h (*p* < 0.05). Pretreatment with *α*-klotho significantly ameliorated acute cardiorenal injury. In the LPS+*α*-klotho (0.01 mg/kg) group, the levels of apoptosis, inflammation, and oxidative stress were decreased, while the level of endoplasmic reticulum stress was elevated.

**Conclusions:**

*α*-Klotho significantly alleviates acute cardiorenal injury in LPS-induced septic cardiorenal injury due to the inhibition of apoptosis, inflammation, and oxidation, as well as the regulation of endoplasmic reticulum stress levels.

## 1. Introduction

Cardiorenal syndrome type 5 (CRS-5) occurs when an overwhelming insult leads to the simultaneous development of acute kidney injury (AKI) and acute cardiac dysfunction [1]. Sepsis is the most important cause of CRS-5 [2]. Sepsis is a dysregulated immune response to an infection that leads to organ dysfunction and is responsible for 9% of deaths every year in developed countries [3, 4]. In sepsis patients, myocardial dysfunction, often termed septic cardiomyopathy, is the most common and severe complication, which presents in nearly 10%~70% of all septic patients and is associated with worse outcomes [5].

There are several mechanisms involved in the pathophysiology of septic cardiomyopathy, including cytokines, nitric oxide (NO), mitochondrial dysfunction, and calcium handling [6]. Exposure of rat cardiomyocytes to the serum of sepsis patients reduced the extent of myocardial cell shortening [7]. Tumor necrosis factor-*α* (TNF-*α*) and IL-1 are responsible for the early cardiac depression, while the prolonged effect of cardiac depression is attributed to excessive myocardial NO synthesis. NO formation by eNOS may contribute to the early myocardial dysfunction in sepsis. The increased expression of iNOS plays a key role in the late cardiac dysfunction associated with sepsis [8].

*α*-Klotho is an antiaging protein predominantly produced in the kidney and several other tissues, including the parathyroid glands and epithelial cells of the choroids plexus [9]. There are two forms of *α*-klotho, including membrane *α*-klotho and secreted *α*-klotho. Membrane *α*-klotho forms a tetrameric complex with fibroblast growth factor (FGF) receptors and functions as a coreceptor for FGF23. Soluble *α*-klotho is a pleiotropic protein that functions as an endocrine factor with renal and extrarenal effects, such as antiaging, antiapoptosis, and antioxidation effects, as well as the modulation of renal iron channels [10].

*α*-Klotho was reported to be protective against chronic cardiovascular and chronic kidney diseases. A previous study found *α*-klotho protected against indoxyl sulfate-induced myocardial hypertrophy via inhibition of oxidative stress and its downstream signaling pathways [11]. *α*-Klotho also inhibited isoprenaline-induced endoplasmic reticulum stress and the apoptotic signal through the suppression of the P38 and Jun N-terminal kinase (JNK) pathway and ultimately improved cardiac pathological changes [12]. In Xie et al.'s study, *α*-klotho-deficient mice developed exaggerated pathological cardiac hypertrophy and remodeling in response to stress, while cardioprotection by *α*-klotho in the control mice is mediated by the downregulation of transient receptor potential cation (TRPC6) channels in the heart [13]. However, the protective effects and underlying mechanisms of *α*-klotho in sepsis-induced acute cardiac injury and acute kidney injury remain unknown. This study aimed to investigate the effects of *α*-klotho in lipopolysaccharide-induced sepsis.

## 2. Materials and Methods

### 2.1. Animal Model

Male 8-week-old C57BL/6 mice (20-24 g) were randomly assigned to 4 groups: the control group (*n* = 6): animals received an intraperitoneal injection of saline (0.5 ml); the LPS group (*n* = 6): animals received an intraperitoneal injection of LPS (10 mg/kg, Sigma-Aldrich) in 0.5 ml of saline; the LPS+*α*-klotho (0.01 mg/kg, R&D) group (*n* = 6): animals received an intraperitoneal injection of recombinant *α*-klotho (0.01 mg/kg) in 0.5 ml of saline 60 min before LPS injection; and the LPS+*α*-klotho (0.02 mg/kg) group (*n* = 6): animals received an intraperitoneal injection of recombinant *α*-klotho (0.02 mg/kg) in 0.5 ml of saline 60 min before LPS injection. Mice were euthanized at 6 h, 24 h, 48 h, and 72 h after LPS treatment. All efforts were made to ameliorate the suffering of animals, and this study was approved by the Ethics Committee of Tongji University.

The expression of *α*-klotho was measured in the mouse heart and kidneys (qPCR and western blotting). The serum troponin, BNP, NGAL, and creatinine were measured in all groups (ELISA). The serum and heart IL-1, IL-6, IL-10, and TNF-*α* levels were measured in the control group, LPS group, and LPS+*α*-klotho (0.01 mg/kg) (ELISA). Malondialdehyde (MDA), reactive oxygen species (ROS), glutathione peroxidase (GSH-Px), superoxide dismutase (SOD), and nitric oxide (NO) in the mouse heart tissue were measured (ELISA). The levels of glucose-regulated protein 78 (GRP78), activating transcription factor (ATF4, ATF6), inositol-requiring enzyme 1 (Ire1), the RNA-like endoplasmic reticulum kinase (Perk), X-box binding protein 1 (XBP1), and C/EBP homologous protein (CHOP) were measured in the mouse heart tissue (qPCR).

### 2.2. Tissue Processing and Histological Examination

At predesigned time points, the mice were euthanized, the hearts and kidneys were carefully collected and washed in cold PBS, and the connective tissues were removed. Each heart was cut into five blocks, three of which were stored in liquid nitrogen for real-time PCR, western blotting, and ELISA and two of which were fixed in 4% formaldehyde for histological examination.

### 2.3. Quantitative Real-Time PCR

Total RNA was extracted from myocardial tissues using TRIzol reagent (Invitrogen). Ultramicro-ultraviolet spectrophotometry (Beckman) was employed to measure the concentration and purity of RNA extracted. cDNA was synthesized from 2 *μ*g of total RNA using a mix of random and oligo(dT) primers with the ReverTra Ace qPCR-RT kit (Toyobo, Osaka, Japan). The average relative mRNA in the control group is defined as 1.0. The mRNA expression was normalized against GAPDH. On the basis of the Ct values of the target genes and internal reference gene, the 2^–ΔΔCt^ method was used to calculate the relative expression of each target gene. All primers are shown in Table 1.

### 2.4. Western Blotting

Proteins were separated by 10% SDS-PAGE and were then transferred onto nitrocellulose membranes. The membrane was washed in TBS-T (0.05% Tween-20 in TBS), blocked with 5% nonfat milk in TBS-T, and incubated with antibodies against *α*-klotho (1 : 1000; Abcam, ab86794), caspase-3 (1 *μ*g/ml; Abcam), or GAPDH (1 : 1000, Santa Cruz) for 12 h. Following three washes with TBS-T, the membrane was incubated with HRP-conjugated secondary antibody for 2 h and then treated with enhanced chemiluminescence detection reagent, followed by autoradiography (Kodak, Rochester, NY, USA). Images were analyzed using ImageJ lab software.

### 2.5. ELISA

The serum and heart tissue samples were measured with enzyme-linked immunosorbent assay kits according to the product protocols (Mlbio, Shanghai). The main steps included the following: (1) standard, sample diluent; (2) add standard, sample diluent, incubate for 30 min at 37°C; (3) wash 5 times, add HRP-conjugated reagent, incubate for 30 min at 37°C; (4) wash 5 times, add chromogen solutions A and B, incubate for 10 min at 37°C; (5) add stop solution; (6) read absorbance at 450 nm within 15 min; and (7) calculate.

### 2.6. Statistical Analysis

SPSS version 21.0 was used for statistical analyses. Data are expressed as the means ± SD. Data were compared with independent sample *t* tests between two groups. A value of *p* < 0.05 was considered statistically significant.

## 3. Results

### 3.1. LPS-Induced Acute Cardiac Injury and Acute Kidney Injury

**Figure 1:**
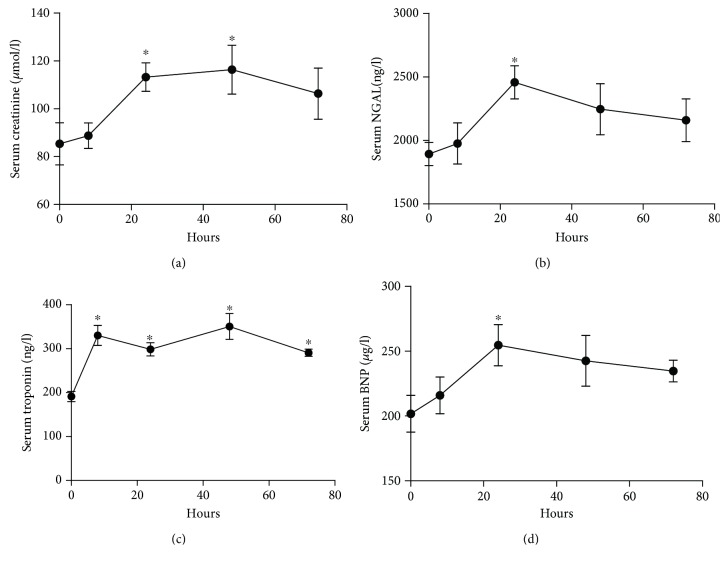
LPS-induced acute cardiac injury and acute kidney injury (a–d). Serum samples were collected from mice at 0 h, 6 h, 24 h, 48 h, and 72 h after. LPS (10 mg/kg) injection (*n* = 6). Serum creatinine (a), NGAL (b), troponin (c), and BNP (d) levels were measured by ELISA. ^∗^*p* < 0.05.

C57BL/6 mice were intraperitoneally injected with LPS at 10 mg/kg and were euthanized at 6 h, 24 h, 48 h, and 72 h after LPS injection. The serum creatinine, NGAL, troponin, and BNP were measured at all time points. As shown in Figure 1, the serum creatinine significantly increased at 24 h (113.0 ± 11.8 vs. 85.2 ± 15.6 *μ*mol/l, *p* < 0.05) and 48 h (116.1 ± 17.6 vs. 85.2 ± 15.6 *μ*mol/l, *p* < 0.05) after LPS injection compared to the control group (Figure 1(a)). The serum NGAL significantly increased at 24 h (2382.2 ± 238.2 vs. 1893.2 ± 156.7 ng/l, *p* < 0.05) after LPS injection compared to the control group (Figure 1(b)). Myocardial injury was evaluated by the serum troponin and BNP. The serum troponin significantly increased as early as 6 h after LPS injection compared to the control group (330.3 ± 45.6 vs. 191.0 ± 20.4 ng/l, *p* < 0.05), and this increase lasted until 72 h (290.8 ± 14.3 vs. 191.0 ± 20.4 ng/l, *p* < 0.05) after LPS injection (Figure 1(c)). The serum BNP significantly increased (247.0 ± 27.2 vs. 201.7 ± 24.6 *μ*g/l, respectively, *p* < 0.05) at 24 h after LPS injection compared to the control group (Figure 1(d)).

### 3.2. LPS Decreased Renal *α*-Klotho Expression

**Figure 2:**
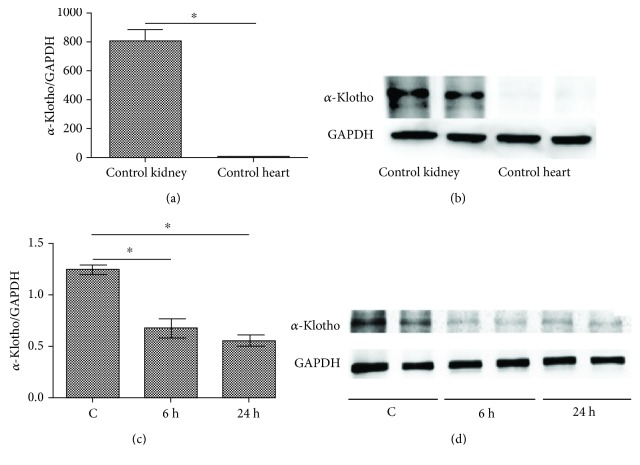
LPS decreased renal *α*-klotho expression (a–d). Mouse heart and kidney tissues were collected from the control group (*n* = 6). Real-time PCR and western blotting analysis showing the expression of *α*-klotho mRNA (a) and protein (b) levels in the control's hearts and kidneys. Mouse kidney samples were collected at 0 h, 6 h, and 24 h after LPS (10 mg/kg) injection in the LPS group (*n* = 6). Real-time PCR and western blotting analysis showing the expression of *α*-klotho mRNA (c) and protein (d) levels in mouse kidneys. ^∗^*p* < 0.05.

The *α*-klotho expression was detected in both control kidneys and hearts. The primers for *α*-klotho and GAPDH mRNA are listed in Table 1. As shown in Figures 2(a) and 2(b), *α*-klotho was undetectable in the control mouse hearts, while it was abundantly expressed in the control mouse kidneys. After LPS injection, the renal *α*-klotho expression markedly decreased as early as 6 h and remained stable until 24 h after LPS injection (Figures 2(c) and 2(d)).

### 3.3. *α*-Klotho Ameliorated LPS-Induced Cardiomyopathy and AKI

**Figure 3:**
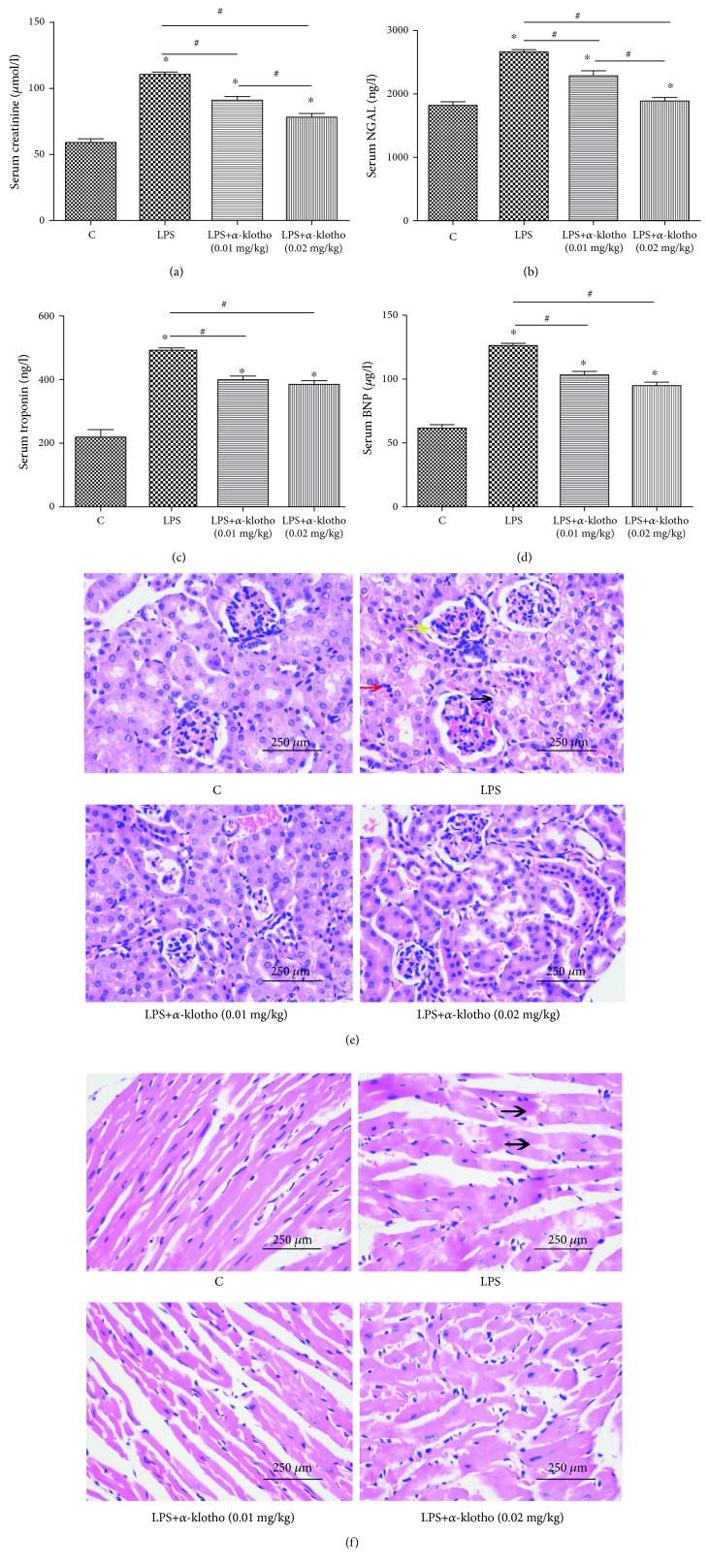
*α*-Klotho alleviated LPS-induced acute cardiac injury and AKI (a–f). Mice were randomly assigned to four groups (*n* = 24): control group, LPS group, LPS+*α*-klotho (0.01 mg/kg) group, and LPS+*α*-klotho (0.02 mg/kg) group. Recombinant *α*-klotho protein was injected an hour before LPS injection. Serum creatinine (a), NGAL (b), troponin (c) ,and BNP (D) levels were measured at 24 h in all groups by ELISA. Heart and kidney tissues were collected for HE staining (400x) at 24 h in four groups. The lesions in kidneys (e) and hearts (f) were marked with different arrows. ^∗^ means *p* < 0.05 compared with the control group. ^#^*p* < 0.05.

We injected recombinant *α*-klotho (0.01 mg/kg or 0.02 mg/kg) an hour before LPS injection to determine whether *α*-klotho exhibits cardiorenal protection. As shown in Figure 3, compared to the LPS group, pretreatment with *α*-klotho significantly decreased troponin (396.2 ± 20.7 vs. 465.3 ± 43.5 ng/l, respectively, *p* < 0.05), BNP (102.8 ± 9.8 vs. 117.8 ± 13.0 *μ*g/l, respectively, *p* < 0.05), creatinine (91.5 ± 7.2 vs. 110.3 ± 3.2 ng/l, respectively, *p* < 0.05), and NGAL (2301.3 ± 186.2 vs. 2645.0 ± 151.8 ng/l, respectively, *p* < 0.05), which demonstrated that *α*-klotho has the ability to alleviate septic cardiorenal injury. Better renoprotective effects of *α*-klotho were achieved at a higher dose (0.02 mg/kg). However, there was no significant difference in the cardioprotection between the *α*-klotho 0.01 mg/kg group and the *α*-klotho 0.02 mg/kg group (Figures 3(a)–3(d)).  Figure 3(e) shows that after LPS injection, the pathological changes were obvious in the kidney and were characterized by mesangial cell proliferation (yellow arrow), vacuolar degeneration of renal tubular epithelial cells (black arrow), and increased inflammatory cells in the renal interstitium (red arrow). In the LPS+*α*-klotho groups, these pathological lesions were alleviated (HE 400x).  Figure 3(f) shows that the pathological changes were obvious in the heart and were characterized by disordered arrangement of myocardial cells and dissolution and fracture of myocardial fibers in the heart (black arrows). Pretreatment with *α*-klotho could alleviate these pathological injuries (HE 400x).

### 3.4. *α*-Klotho Reduced LPS-Induced Apoptosis in Mouse Heart

**Figure 4:**
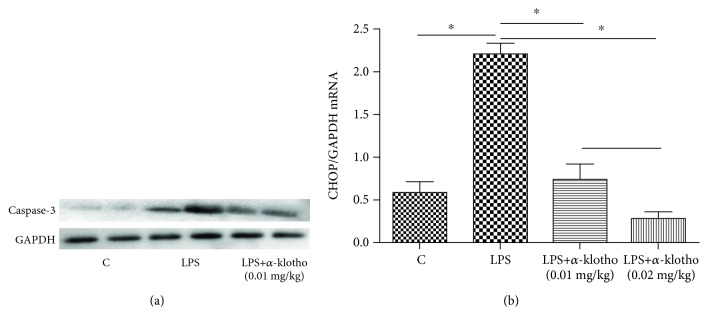
*α*-Klotho reduced LPS-induced apoptosis in mouse heart (a, b). Mouse heart tissues were collected from the control group, LPS group, and LPS+*α*-klotho (0.01 mg/kg) group at 24 h after LPS injection. The levels of heart caspase-3 (a) were measured by western blotting. The expression of heart CHOP mRNA (b) was measured by real-time PCR. ^∗^*p* < 0.05.

As shown in Figure 4(a), LPS significantly increased caspase-3 compared to the control group at 24 h after LPS injection, while *α*-klotho (0.01 mg/kg) pretreatment significantly reduced caspase-3.  Figure 4(b) shows that CHOP expression increased in the LPS group, while it was markedly reduced in the LPS+*α*-klotho group (*p* < 0.05 vs. LPS group), which indicates that *α*-klotho can inhibit cardiomyocyte apoptosis. The primers for CHOP and GAPDH mRNA are listed in Table 1.

### 3.5. *α*-Klotho Regulated Cytokines in Sepsis

**Figure 5:**
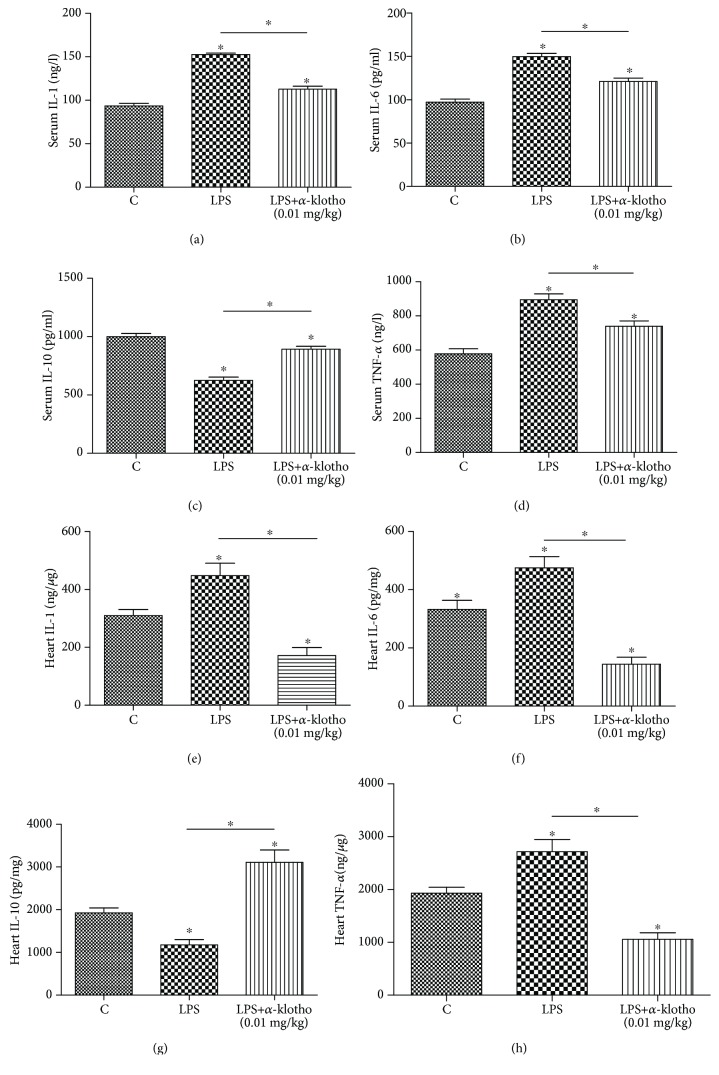
*α*-Klotho regulated cytokines in sepsis (a–h). Heart tissues and serum samples were collected at 24 h after LPS injection in the control group (*n* = 6), LPS group (*n* = 6), and LPS+*α*-klotho (0.01 mg/kg) group (*n* = 6). The levels of IL-1 (a, e), IL-6 (b, f), IL-10 (c, g), and TNF-*α* (d, h) were measured by ELISA. ^∗^*p* < 0.05.

As shown in Figure 5, the injection of LPS increased proinflammatory cytokines, such as IL-1, IL-6, and TNF-*α*, while it decreased the anti-inflammatory cytokine IL-10, compared with the levels in the control group (*p* < 0.05). As shown in Figures 5(a)–5(d), the serum IL-1 (152.6 ± 5.7 vs. 93.4 ± 11.0 ng/l, *p* < 0.05), IL-6 (149.8 ± 13.3 vs. 97.3 ± 11.8 pg/ml, *p* < 0.05), and TNF-*α* (894.9 ± 118.0 ng/l vs. 577.5 ± 103.7, *p* < 0.05) significantly increased in the LPS group compared to the levels in the LPS+*α*-klotho (0.01 mg/kg) group, while the serum IL-10 significantly decreased compared to the levels in the LPS+*α*-klotho (0.01 mg/kg) group (624.6 ± 97.5 vs. 999.7 ± 94.5 pg/ml, *p* < 0.05). The same tendencies were also observed in the mouse heart (Figures 5(e)–5(h)). *α*-Klotho (0.01 mg/kg) significantly reduced the mouse heart tissue proinflammatory cytokines compared to those in the LPS group: IL-1 (112.7 ± 12.5 vs. 152.6 ± 5.7 ng/l, *p* < 0.05), IL-6 (121.0 ± 14.1 vs. 149.8 ± 13.3 pg/ml, *p* < 0.05), and TNF-*α* (739.2 ± 108.1 vs. 894.9 ± 118.0 ng/l, *p* < 0.05); moreover, *α*-klotho markedly elevated the anti-inflammatory cytokine IL-10 (892.0 ± 87.0 vs. 624.6 ± 97.5 pg/ml, *p* < 0.05).

### 3.6. *α*-Klotho Alleviates LPS-Induced Oxidative Stress

**Figure 6:**
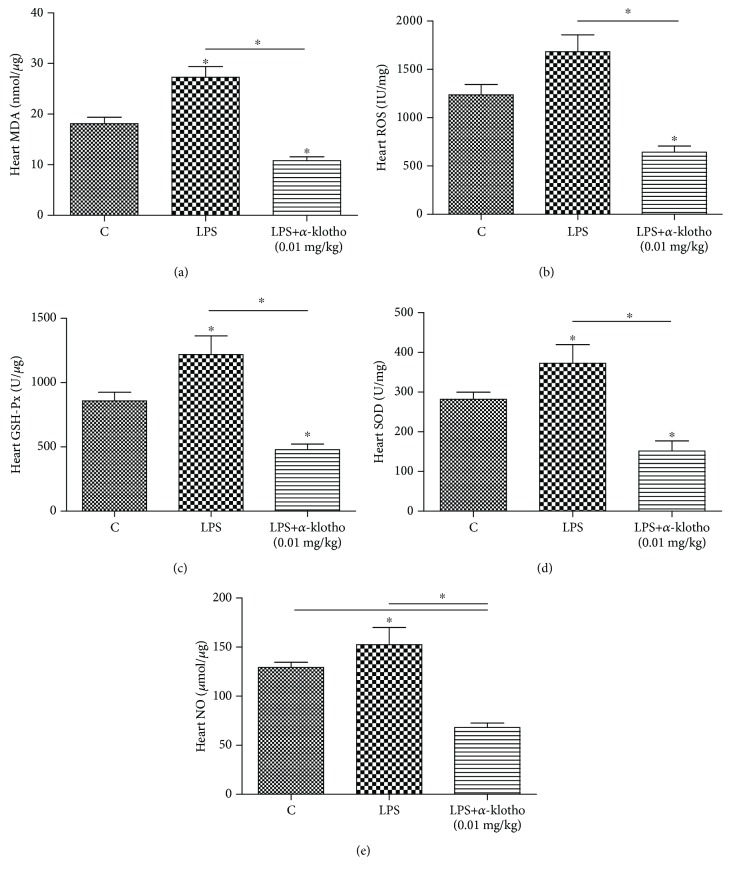
*α*-Klotho reduced LPS-induced oxidative stress in mouse heart (a–e). Heart tissues were collected at 24 h after LPS injection in the control group (*n* = 6), LPS group (*n* = 6), and LPS+*α*-klotho (0.01 mg/kg) group (*n* = 6). The mRNA levels of MDA (a), ROS (b), GSH-Px (c), SOD (D), and NO (e) were measured by ELISA. ^∗^*p* < 0.05.

The oxidative stress was assessed in the mouse heart. As shown in Figure 6, compared to the control group, the heart MDA concentration significantly increased in the LPS group. *α*-Klotho (0.01 mg/kg) pretreatment was able to lower MDA in the mouse heart compared to the LPS group (10.8 ± 2.6 vs. 27.3 ± 7.3 nmol/*μ*g, respectively, *p* < 0.05). There was no significant difference in the heart ROS between the control group and the LPS group; however, the level in the LPS+*α*-klotho (0.01 mg/kg) group was significantly lower than that in the LPS group (642.7 ± 155.4 vs. 1681.9 ± 430.3 IU/mg, respectively, *p* < 0.05). *α*-Klotho (0.01 mg/kg) pretreatment also significantly reduced the heart GSH-Px (477.6 ± 108.8 vs. 1217.2 ± 353.6 U/*μ*g, respectively, *p* < 0.05 vs. LPS group) after LPS treatment. The heart SOD significantly increased after LPS treatment (*p* < 0.05 vs. control group), while it was substantially reduced in the presence of *α*-klotho (0.01 mg/kg) pretreatment (372.8 ± 114.1 vs. 151.7 ± 61.8 U/mg, respectively, *p* < 0.05 vs. LPS group). Compared with the control and LPS groups, NO was significantly reduced in the LPS+*α*-klotho (0.01 mg/kg) group (129.3 ± 5.2 vs. 152.4 ± 42.7 vs. 68.0 ± 11.0 *μ*mol/*μ*g, respectively, *p* < 0.05).

### 3.7. *α*-Klotho Increased ER Stress in Mouse Heart in Sepsis

**Figure 7:**
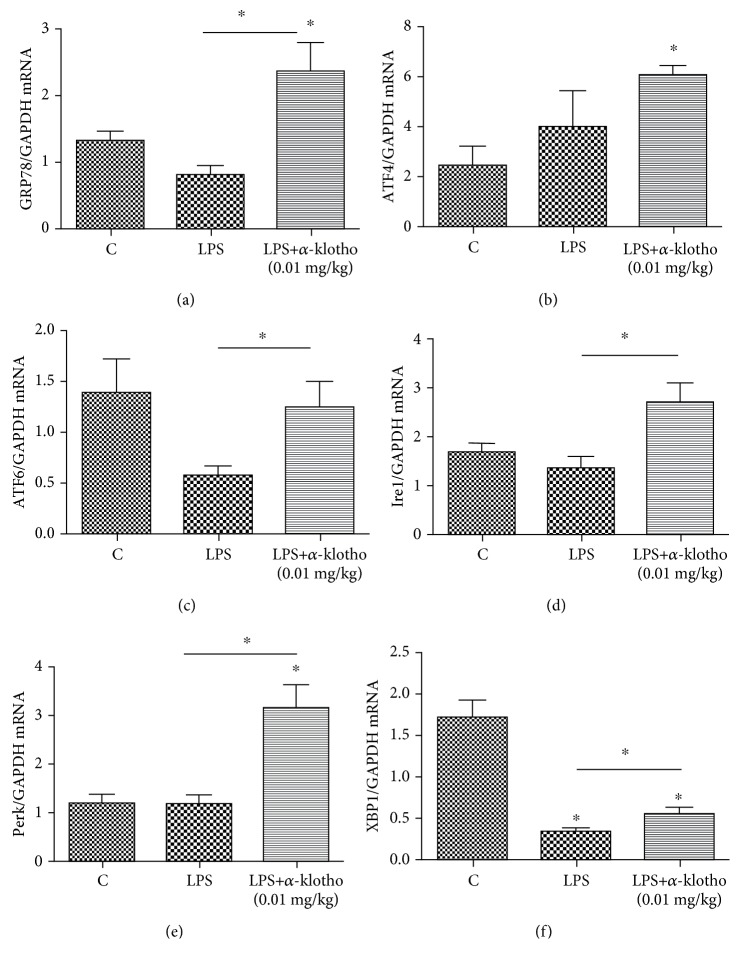
*α*-Klotho elevated the mRNA expression of ER stress-related proteins in LPS-stimulated mouse heart (a–f). Heart tissues were collected at 24 h after LPS injection in the control group (*n* = 6), LPS group (*n* = 6), and LPS+*α*-klotho (0.01 mg/kg) group (*n* = 6). The mRNA expressions of GRP78 (a), ATF4 (b), ATF6 (c), Ire1 (d), Perk (e), and XBP1 (f) were measured at 24 h after LPS injection by real-time PCR. ^∗^*p* < 0.05.

The expression of ER stress biomarkers (GRP78, ATF4, ATF6, Ire1, Perk, and XBP1) was detected in the different groups (Figures 7(a)–7(f)). The primers for GRP78, ATF4, ATF6, Ire1, Perk, XBP1, and GAPDH mRNA are listed in Table 1. Real-time PCR showed the mRNA expression of GRP78, ATF4, ATF6, Ire1, and Perk significantly increased in the *α*-klotho-pretreatment group compared to the LPS group. XBP1 was lower in both the LPS group and LPS+*α*-klotho (0.01 mg/kg) group, and there was a significant difference between the two groups. These findings suggest that *α*-klotho pretreatment may increase ER stress in the mouse heart.

## 4. Discussion

In this study, LPS was used to induce septic cardiorenal syndrome type 5 animal models. In our preliminary experiments, we intraperitoneally injected doxorubicin, cisplatinum, and LPS to induce cardiorenal injury in mice. In the doxorubicin group, heart failure and renal failure were observed at 8 weeks. However, it required a long time to establish the model. Furthermore, the mortality was as high as 31.4%. In the cisplatinum group, acute kidney injury was observed at 5 days; however, the cardiac injury was very mild. In the LPS group, acute cardiac injury and acute kidney injury were observed at 6 h and 24 h, respectively, after injection. Due to its simplicity, feasibility, high success rate, and low mortality, we ultimately chose LPS to establish the septic CRS-5 models.

Clinical trials and animal studies have shown that sepsis may lead to acute heart injury and acute kidney injury, which refer to septic cardiomyopathy and septic AKI, respectively. In our study, after injection of LPS, the serum troponin level was increased at 6 h and remained stable until 72 h, while the serum creatinine, NGAL, and BNP levels were significantly increased at 24 h, which indicates the successful establishment of septic CRS-5 animal models.

Previous studies showed that *α*-klotho mRNA was expressed in multiple tissues, including the heart, aorta, colon, pituitary gland, thyroid gland, pancreas, and gonads; however, the strongest expression by far is in the kidney [14]. In our study, we detected the *α*-klotho expression in mouse hearts and kidneys. The results show that both mRNA and protein levels of *α*-klotho in mouse hearts were undetectable, while the expressions were very high in the mouse kidney. Recent studies have found that the renal *α*-klotho expression was reduced in patients with sepsis-induced acute kidney injury, while renal damage markers, such as NGAL and KIM-1, were increased [15], which is consistent with our findings. In our studies, after injection of LPS, the levels of *α*-klotho in the kidneys were significantly reduced as early as 6 h. The decrease in renal *α*-klotho has also been shown in several other animal models, such as renal ischemia-reperfusion injury [16], the db/db mouse model of diabetes [17], the unilateral ureteral ligation (UUO) model [18], and the cisplatin-induced AKI model [19], which indicates that the kidney was the main source of *α*-klotho. When kidney injury occurred, the *α*-klotho expression was generally reduced. The mechanism by which *α*-klotho expression decreases in these animal models remains unclear. It was reported that inflammatory cytokines such as TWEAK and TNF-*α* could downregulate *α*-klotho expression through an NF-*κ*B-dependent mechanism [20, 21]. As a systemic inflammatory response to infection, various inflammatory factors significantly increase in cases of sepsis, and these inflammatory cytokines may contribute to the reduced klotho expression in the kidney.

Previous studies showed that *α*-klotho deficiency may aggravate heart and kidney injuries when exposed to damage, while supplementation of *α*-klotho could attenuate AKI and stress-induced cardiac hypertrophy [11, 13, 16]. Recent studies showed that the administration of recombinant *α*-klotho protects against LPS-induced kidney damage and attenuates LPS-mediated endothelial activation [15]. In our study, pretreatment with *α*-klotho significantly ameliorates acute cardiorenal injury. As shown in Figure 3, the serum creatinine, NGAL, BNP, and troponin levels were all reduced in the *α*-klotho pretreatment groups. Furthermore, the kidney recovery was more remarkable in the high-dose group. However, there was no significant reduction in serum BNP and troponin between the low-dose and high-dose *α*-klotho pretreatment groups.

Our study showed that *α*-klotho pretreatment significantly improved septic cardiorenal injury. However, the mechanism that underlies the protective effects of *α*-klotho on LPS-induced cardiorenal injury remains unclear. Many factors might be involved in the protective effects of *α*-klotho.

It has been shown that *α*-klotho was able to ameliorate the apoptosis of renal tubule cells in an ischemic acute renal failure model, accompanied by improved kidney function and tubulointerstitial injury in a hypertension model [22, 23]. In addition, *α*-klotho may inhibit isoproterenol-induced cardiomyocyte apoptosis [12]. In our study, *α*-klotho was shown to remarkably decrease the caspase-3 and CHOP levels after LPS injection, which indicates that *α*-klotho inhibits LPS-induced cardiomyocyte apoptosis.

Another mechanism involved is related to the anti-inflammatory activity of *α*-klotho. Sepsis is associated with abnormal host immunity in response to pathogen exposure, including exposure to endotoxin. Cytokines play crucial roles in the initiation and resolution of inflammation in sepsis. Recent studies showed that pretreatment with *α*-klotho decreased the renal cytokine IL-6, IL-8, and MCP levels [15]. In patients with CKD, the resolution of inflammation secondary to intercurrent septic processes goes along with a doubling in the levels of circulating iFGF23 and a downregulation of *α*-klotho. iFGF23 downregulation and *α*-klotho upregulation may participate in the response to acute inflammation/sepsis in patients with CKD [24]. The above findings are consistent with our research. Furthermore, our study not only observed proinflammatory factors (IL-1, IL-6, and TNF-*α*) but also anti-inflammatory cytokine IL-10. In our study, *α*-klotho significantly decreased the serum IL-1, IL-6, and TNF-*α* levels and markedly elevated the serum IL-10. This finding indicates that *α*-klotho pretreatment may improve the expression of both pro- and anti-inflammatory cytokines in response to LPS exposure. The anti-inflammatory effects of *α*-klotho have also been studied in other models, such as in spontaneous hypertensive rats [25]. Our findings confirmed the anti-inflammatory activity of *α*-klotho: it can decrease proinflammatory factors and increase anti-inflammatory factors, thus shifting the balance towards an anti-inflammatory environment.

The heart requires significant amounts of energy to sustain its continuous contractile activity. The mitochondria occupy between 22 and 37% of the cardiomyocyte volume across different mammalian species. Mitochondrial dysfunction plays a significant role in the pathogenesis of sepsis [26]. In addition to their role in ATP production, the mitochondria also play an essential role in numerous other cell functions, such as calcium homeostasis, reactive oxygen, and nitrogen species production, and cell signaling and they are key regulators of apoptosis and cell death. Mitochondria are the primary source of ROS within cells. When mitochondrial dysfunction persists in the case of sepsis, ROSs are generated in cardiomyocytes, and the oxidative stress induced by ROS may further cause the deterioration of mitochondrial injury, thereby accelerating mitochondrial dysfunction. In patients with sepsis, 216 dysregulated mitochondrial genes were detected in septic hearts. Overall, 198 of these genes, including the Krebs (TCA) cycle and electron transport components, decreased by 43 ± 5% (mean ± s.d.) [27]. In this study, the injection of LPS increased oxidative stress, while pretreatment with *α*-klotho substantially decreased oxidative stress biomarkers, including MDA, ROS, SOD, GSH-Px, and NO, which suggests that *α*-klotho may play a role as an antioxidative stress factor.

The endoplasmic reticulum (ER) is a subcellular organelle responsible for protein folding and assembly and is involved in several other physiological activities. Under stress and inflammation conditions, ER dysfunction may be present, which is also termed ER stress [28]. However, in our study, the biomarkers of ER stress, GRP78 and XBP1, significantly decreased after LPS treatment. Most notably, *α*-klotho pretreatment significantly elevated all the ER stress biomarkers (GRP78, ATF4, ATF6, Ire1, Perk, and XBP1). In the study of Wei et al., *α*-klotho downregulated GRP78 in human umbilical vein endothelial cells [29], which is inconsistent with our findings. Thus, the effect of *α*-klotho on ER stress in different cells and models requires further studies.

In conclusion, we established a successful septic CRS-5 animal model in mice. LPS significantly decreased *α*-klotho expression in the kidney. *α*-Klotho deficiency may aggravate septic cardiomyopathy and septic AKI. In contrast, pretreatment with *α*-klotho could attenuate LPS-induced cardiorenal injury. The cardiorenal protective function of *α*-klotho may involve its antiapoptosis, anti-inflammation, and antioxidative stress effects. Our study also found that *α*-klotho may elevate the level of ER stress, which needs more studies to confirm.

There is one limitation in our study. As *α*-klotho was administrated an hour before LPS injection, its therapeutic effect in sepsis could not be directly proven. However, in previous studies with the administration of *α*-klotho after renal ischemia-reperfusion experiments, the renal protection of the *α*-klotho protein could also be observed. We can perform further studies to prove the therapeutic effect of *α*-klotho in septic cardiorenal syndrome type 5.

## Figures and Tables

**Table 1:** Sequence of primer pairs for qPCR.

Name	Forward primer (5′-3′)	Reverse primer (5′-3′)
GAPDH	GGCAAGTTCAACGGCACA	CCATTTGATGTTAGCGGGAT
*α*-Klotho	TCCATCTGGGACACTTTCAC	TAACTATCGCTGGCCACATC
ATF4	CCTTCGACCAGTCGGGTTTG	CTGTCCCGGAAAAGGCATCC
ATF6	TCGTGTTCTTCAACTCAGCAC	TGGAGTCAGTCCATGTTCTGT
Grp78	ACTTGGGGACCACCTATTCCT	ATCGCCAATCAGACGCTCC
Ire1	ATGGCTACCATTATCCTGAGCA	TCCTGGGTAAGGTCTCCGTG
Perk	TGTCTTGGTTGGGTCTGATG	TCCTTCTTGCGGATGTTCTT
XBP1	TCCGCAGCACTCAGACTAC	GTTCCTCCAGATTAGCAGACTC
CHOP	GCCGGAACCTGAGGAGAGAGTGT	ACTCAGCTGCCATGACTGCACG

## Data Availability

The data used to support the findings of this study are available from the corresponding author upon request.
